# *Acta Crystallographica Section C*: Chemistry Matters

**DOI:** 10.1107/S2053229623000220

**Published:** 2023-01-12

**Authors:** Paul R. Raithby

**Affiliations:** a Department of Chemistry, University of Bath, Bath BA2 7AY, United Kingdom

**Keywords:** editorial, crystallography, chemistry

## Abstract

Chemistry matters to *Acta Crystallographica Section C*. Authors are encouraged to present new chemistry where there is a clear chemical story based around molecular structure. In this context, ‘structure’ is used loosely and is not in any way restricted to structures determined by single-crystal X-ray crystallography, but includes those determined from powder X-ray or neutron data and NMR spectroscopy, and through theoretical calculations.

As I come towards the end of my term as one of the Main Editors of *Acta Crystallographica Section C*, it is appropriate to take this opportunity at the beginning of a new calendar year to provide readers with a short update on the progress that the journal has been making. I would also like to thank everyone who has contributed to the journal, including all the authors, referees, the Managing Editor and the team in the Chester Editorial Office, the Co-editors and my fellow Main Editors. It also provides me with the opportunity to welcome our two new Main Editors, Amy Sarjeant from Bristol-Myers Squibb Company in the USA and Alan Kennedy from the University of Strathclyde in the UK. They have been in post since October and are already bringing new ideas for the future development of *Acta Crystallographica Section C*.

Over the last decade *Acta Crystallographica Section C* has been continuing to transition to a journal that publishes exciting new structural science with an emphasis on mol­ecular chemistry. Authors are encouraged to present new chemistry where there is a clear chemical story based around mol­ecular structure. In this context, ‘structure’ is used loosely and is not in any way restricted to structures determined by single-crystal X-ray crystallography, but includes those determined from powder X-ray or neutron data and NMR spectroscopy, and through theoretical calculations. Structures determined using Hirshfeld atom refinement and related methods are also encouraged.

The journal continues to highlight exciting aspects of structural chemistry through feature articles and special issues. The next special issue is on *noncovalent inter­actions based on the sigma-hole*, that my co-Main Editor, Jonathan White, is organising with Professor Lee Brammer and two members of his group, from the University of Sheffield, as guest editors. This is a highly topical area of structural research with the importance of these noncovalent interactions in a broad range of chemistry only now being fully appreciated. The publication date will be early in 2023. Further special issues will follow later in 2023 and 2024.

I would like to thank the contribution made by the retiring Co-editors Yoshiki Ohgo, Tong-Bu Lu, Chris Frampton and Eugene Cheung, and my particular thanks go to my co-Main Editor, Larry Falvello, who will be retiring in 2023. His contribution to the journal over many years has been enormous, and I am most grateful to him for the scientific rigour and good common sense that he applies to all matters relating to *Acta Crystallographica Section C*, and for the patience and kindness that he has always shown to me and the other editors and contributors to the journal. Finally, I would like to acknowledge all the hard work, support and friendship of the Chester Editorial Office staff during my time as a Main Editor. It is ultimately they who get the accepted manuscripts through production into the final product that we can all read and enjoy.

